# Femorotibial rotation does not affect clinical outcomes after patellofemoral stabilizing surgery

**DOI:** 10.1002/jeo2.70532

**Published:** 2025-11-14

**Authors:** Marc Schmid, Giuseppe Loggia, Andreas Flury, Gabriele Cirigliano, Stefan Zimmermann, Lazaros Vlachopoulos, Sandro Hodel, Sandro Fucentese

**Affiliations:** ^1^ Department of Orthopedics Balgrist University Hospital University of Zurich Zurich Switzerland

**Keywords:** femorotibial rotation, knee rotation, patellar instability, winking sign

## Abstract

**Purpose:**

The role of femorotibial rotation in patellar instability treatment and prognosis remains unclear. This study examines whether increased femorotibial rotation, indicated by a positive winking sign, affects functional outcomes and recurrent instability after patellofemoral stabilizing surgery.

**Methods:**

All patients undergoing patellofemoral instability surgery at our institution (2014–2022) with complete rotational imaging (magnetic resonance imaging/computed tomography [MRI/CT]) and functional assessments (Kujala, Tegner) and at least 1‐year follow‐up were included. Patients were grouped based on the presence of a radiological winking sign. Surgical treatment was tailored to individual deformities, including medial patellofemoral ligament reconstruction, trochleoplasty, derotational osteotomy and tibial tuberosity osteotomy.

**Results:**

A total of 121 knees (114 patients, mean age 23.5 years) with a mean follow‐up of 2 years were analyzed. Preoperatively, the winking sign was present in 19.8% (*n* = 24) and absent in 80.2% (*n* = 97). Demographics and preoperative deformity analysis were similar between groups (n.s.) except for increased femorotibial rotation (11.8° ± 7° vs. 8.5° ± 5°; *p* = 0.031) and patellar tilt (*p* = 0.006) in patients with a positive winking sign. Functional outcome scores either improved (Kujala: 68.9 ± 16 to 80.8 ± 19; *p* < 0.001) or remained unchanged (Tegner: 3.6 ± 1.4 to 3.4 ± 1.6; *p* = 0.347) from pre‐ to postoperative. Patients with a positive winking sign tended to show less improvement (Δ Kujala: 7.6 ± 18 vs. 13.0 ± 20; *p* = 0.170; Δ Tegner: 0.0 ± 1.7 vs. −0.1 ± 2.0; *p* = 0.373). Surgical procedures were evenly distributed between groups (n.s.). One patient (0.8%) with a negative winking sign had a recurrent instability. Complications did not differ between groups (n.s.).

**Conclusion:**

Patients with increased femorotibial rotation achieve similar functional outcome following patellofemoral stabilizing surgery without increased complications or persistent instability. A tendency for less functional improvement in these patients raises questions about the need to assess femorotibial rotation as an independent deformity. Further research is needed to investigate this topic more deeply.

**Level of Evidence:**

Level III.

**Abbreviations**
3D: 3‐dimensional
BMI: body mass index
CT: computed tomography
HKA: hip–knee–ankle angle
MPFL: medial patellofemoral ligament
MRI: magnetic resonance imaging
n.s.: not significant
PROMs: patient‐reported outcome measures
SD: standard deviation
SPSS: Statistical Package for the Social Sciences
TT: tibial tuberosity
TTO: tibial tuberosity osteotomy
TT‐TG: tibial tuberosity‐trochlear groove

## INTRODUCTION

Understanding bony malalignment and the correlation between bony parameters is crucial for treating patellofemoral disorders. Various parameters have been described and implicated in patellofemoral instability, depending on the bony geometry. In addition to well‐known factors such as femoral and tibial torsion, trochlear dysplasia, frontal mechanical axis, tibial tuberosity‐trochlear groove (TT‐TG) distance, recent studies raised awareness on the relative rotation of the femur on the tibia and its role in the pathophysiology of patellar instability [11, 17, 18].

**Figure 1:**
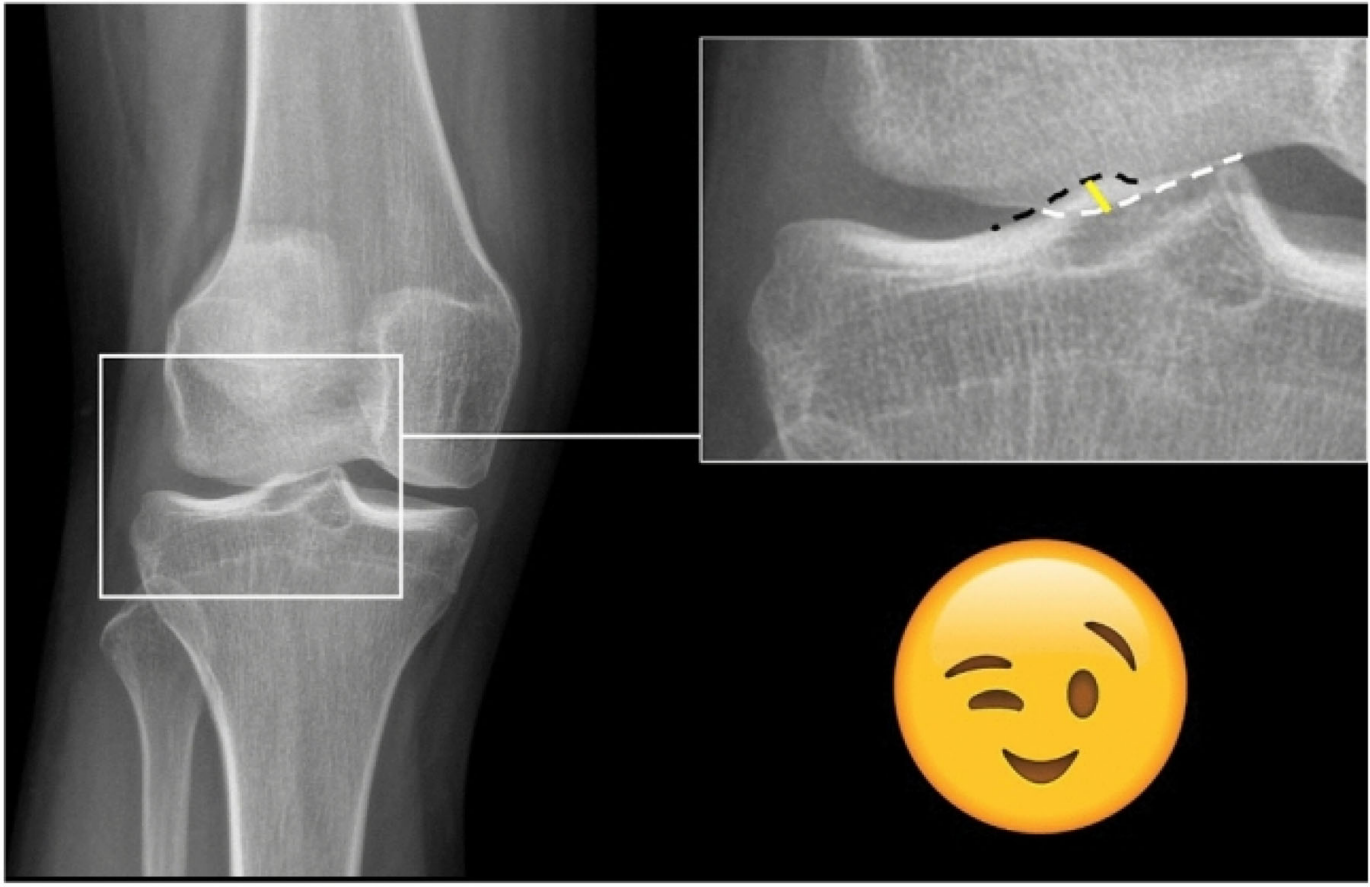
Definition of the winking sign and measurement of the overlap of the lateral femoral condyle and the lateral tibial eminence. Left: Example of a positive winking sign in a female patient with femorotibial torsion of 22°. Right (magnification): An overlap of the femoral condyle (white dotted line) with the lateral tibial eminence (black dotted line) defined a positive winking sign. The femoral condyle overlap was measured perpendicular to the lateral tibial eminence at the location of the greatest overlap (yellow line) in mm [6].

A recent study introduced the winking sign, a simplified method for diagnosing femorotibial malrotation using conventional weight‐bearing radiographs of the knee [6]. The winking sign describes an overlap of the lateral femoral condyle and the lateral tibial eminence. The presence of such an overlap indicates an average femorotibial rotation of 6.3°, while an overlap greater than 2 mm suggests a femorotibial rotation of more than 15°, as shown in Figure 1 [6]. However, the dynamic impact and functional relevance of increased femorotibial rotation remain unclear. Additionally, there is no consensus on what constitutes normal versus increased femorotibial rotation.

The relationship to other morphological findings is evident, as increased femorotibial rotation is commonly associated with patellar tilt and TT‐TG distance, both of which are primarily influenced by knee rotation [2, 21]. Additionally, it remains unclear whether increased femorotibial rotation significantly impacts clinical outcomes. This raises the question of whether femorotibial rotation should be corrected during patellar stabilization procedures, and more importantly, how this issue can effectively be addressed surgically. Currently, there is limited knowledge on how to correct femorotibial rotation and how different surgical techniques might influence this condition.

The primary aim of this study is to investigate the potential impact of preoperative femorotibial rotation on postoperative patient‐reported outcome measures (PROMs) as well as functional outcomes such as recurrent instability and revision operations. By evaluating this correlation, the aim is to define whether femorotibial rotation prior to surgery has any significant influence on the overall outcome. Additionally, the relationship between the outcome and both femorotibial rotation observed in three‐dimensional (3D) nonweight‐bearing imaging and the presence of a winking sign detected in plain radiographs is to be investigated (Figure 1).

We hypothesized that a positive winking sign on preoperative radiographs is associated with worse clinical outcomes after patellofemoral stabilizing surgery.

## METHODS

This study was approved by the Institutional Review Board and ethical approval was obtained from the local ethics committee, and written informed consent was obtained from all study participants. It was conducted entirely at the authors' institution.

### Study cohort

**Figure 2:**
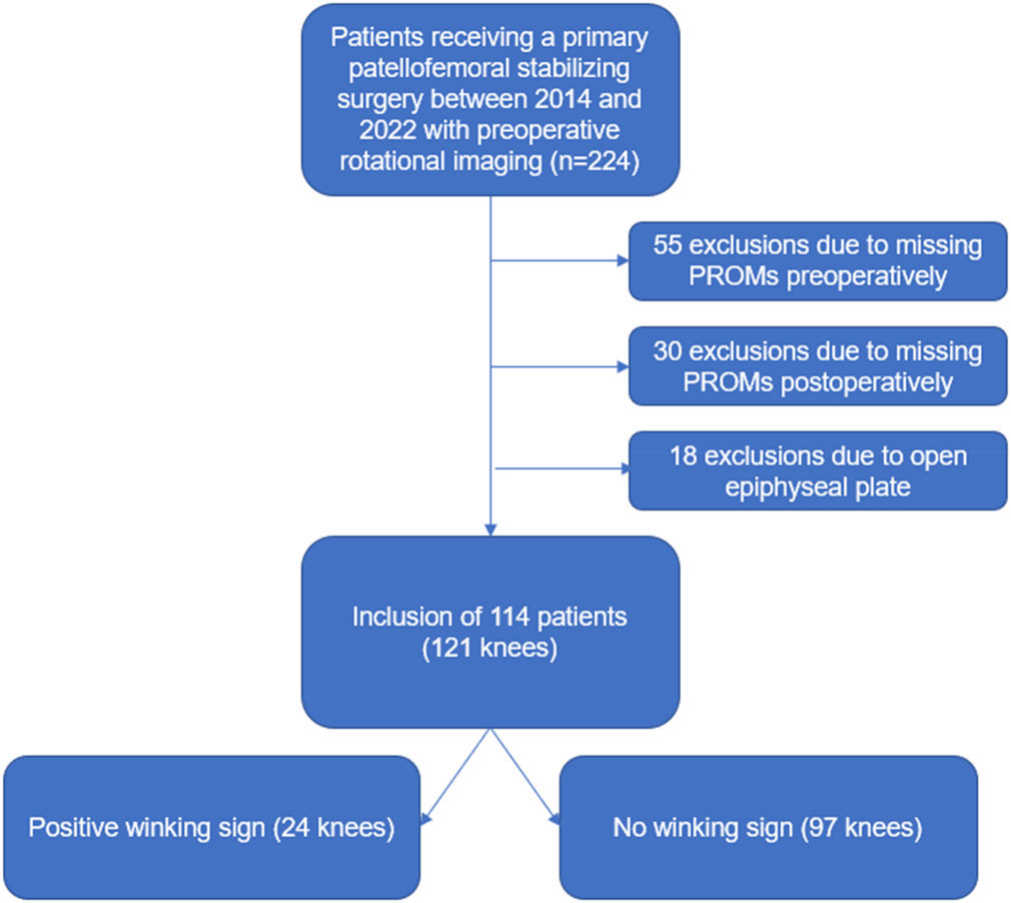
Flowchart for eligibility. PROMs, patient‐reported outcome measures.

This retrospective single‐center study involving patients who underwent primarily patellofemoral stabilizing surgery between January 2014 and December 2022, with a minimum follow‐up of 1 year, was conducted. From an initial patient cohort, comprising 224 patients who had undergone preoperative 3D imaging to assess leg torsion, individuals missing preoperative (*n* = 55) or postoperative (*n* = 30) PROMs were excluded. Additionally, individuals with open epiphyseal plates (*n* = 18) were also excluded. Ultimately, our analysis included a final sample size of 121 knees from 114 patients (Figure 2). Based on our sample size, patients were categorized into two groups according to the presence or absence of the winking sign.

### Surgical technique

The surgical approach was tailored to address the individual deformities of each patient. The treatment included a series of procedures aimed to restore optimal knee function and stability. Among these interventions, reconstruction of the medial patellofemoral ligament (MPFL) was undertaken according to Schöttle [20] to address patellar instability and insufficient medial soft tissue restraint, while trochleoplasty was performed with the Bereiter technique [1] and aimed at reshaping the trochlear groove for improved patellar tracking in patients with trochlear dysplasia Dejour type B–D. Additionally, femoral and/or tibial derotational osteotomy procedures were utilized to correct rotational malalignment within the knee joint (Femoral osteotomy for excessive femoral antetorsion (>25°) and/or genu varum or valgum, tibial osteotomy for excessive external tibial torsion (>35°) and/or genu varum or valgum). Finally, tibial tuberosity osteotomy (TTO) was considered when TT‐TG was increased (>15 mm) to realign the extensor mechanism. By integrating these surgical techniques, our approach aimed to address the multifactorial nature of each patient's condition, ultimately striving to achieve optimal functional outcomes and long‐term joint health.

### Demographics and scores

In addition to gathering demographic information such as age, gender and body mass index (BMI) from patient records, functional outcomes using validated scoring systems such as the Kujala score [16] and the Tegner score [23] both pre‐ and postoperatively to assess knee function and its impact on the daily activities were evaluated. Finally, the patient records were searched to identify any reoperations, redislocations or persistent instability documented in postoperative reports.

### Radiographic measurements

Before the surgical procedure, all patients underwent supine rotational computed tomography (CT) scans or magnetic resonance imaging (MRI) scans of the affected limb. These scans were conducted according to a standardized protocol, including the complete knee joint, alongside providing additional insight into the hip and ankle joints.

The hip–knee–ankle angle (HKA) [3, 7], femorotibial torsion [18] (the rotational relationship between the femur and tibia, measured from the posterior condylar axis to the proximal tibial plateau), as well as femoral [9, 14, 22] (the axial rotation of the femur, measured from the femoral neck axis to the posterior condylar axis) and tibial torsion [8, 10, 13] (the rotational alignment of the tibia, measured from the proximal tibial plateau to the distal bimalleolar axis), TT‐TG distance [5, 10, 13], patellar tilt [5], trochlear dysplasia [4] and finally the presence of a winking sign in weight‐bearing X‐rays [6] were measured according to well‐known and prior defined measuring methods.

All measurements were conducted by two trained observers. To enhance the robustness of our analysis, the mean value from the measurements of both observers was used for statistical calculations.

Additionally, an analysis of interobserver agreement was performed to assess the consistency between the measurements taken by the two observers.

### Statistics

The sample size was determined based on the available cases, as outlined in the methods.

The normality of the data distribution was assessed using the Shapiro–Wilk test. The data are presented as mean ± standard deviation (SD) or as counts (percentages). Continuous variables between patients with and without a winking sign were analyzed using an unpaired Student's *t*‐test or the Mann–Whitney *U* test, as appropriate. Differences between categorical variables were analyzed using Pearson's *χ* ^2^ test. To assess the interrater reliability of angle measurements between two fixed raters, an intraclass correlation coefficient (ICC) was calculated using a two‐way mixed‐effects model for absolute agreement of single measurements, denoted as ICC (3,1). The level of significance for all statistical tests was set at <0.05. All data analyses were performed using SPSS version 29 (SPSS Inc.).

## RESULTS

The final analysis included a cohort of 121 patients, with a mean age of 23.5 years (range: 14–48 years). Within this cohort, the majority were women (70.2%), the mean BMI was 25.2 kg/m^2^ (range: 17.6–42.0). The average follow‐up duration was 2 years, ranging from 1 to 9 years (Table 1).

**Table 1:** Demographics.

	Positive winking sign (*n* = 24)	No winking sign (*n* = 97)	*p* value
Age (years)	23.9 ± 6.3	23.4 ± 8.3	n.s.
Female	20 (83%)	65 (67%)	n.s.
Male	4 (17%)	32 (33%)	n.s.
BMI (kg/m^2^)	23.6 ± 3.4	26.0 ± 5.4	n.s.

Abbreviation: BMI, body mass index.

The study population was divided into two groups: patients with a positive winking sign (*n* = 24) and patients without a winking sign (*n* = 97) preoperatively.

**Figure 3:**
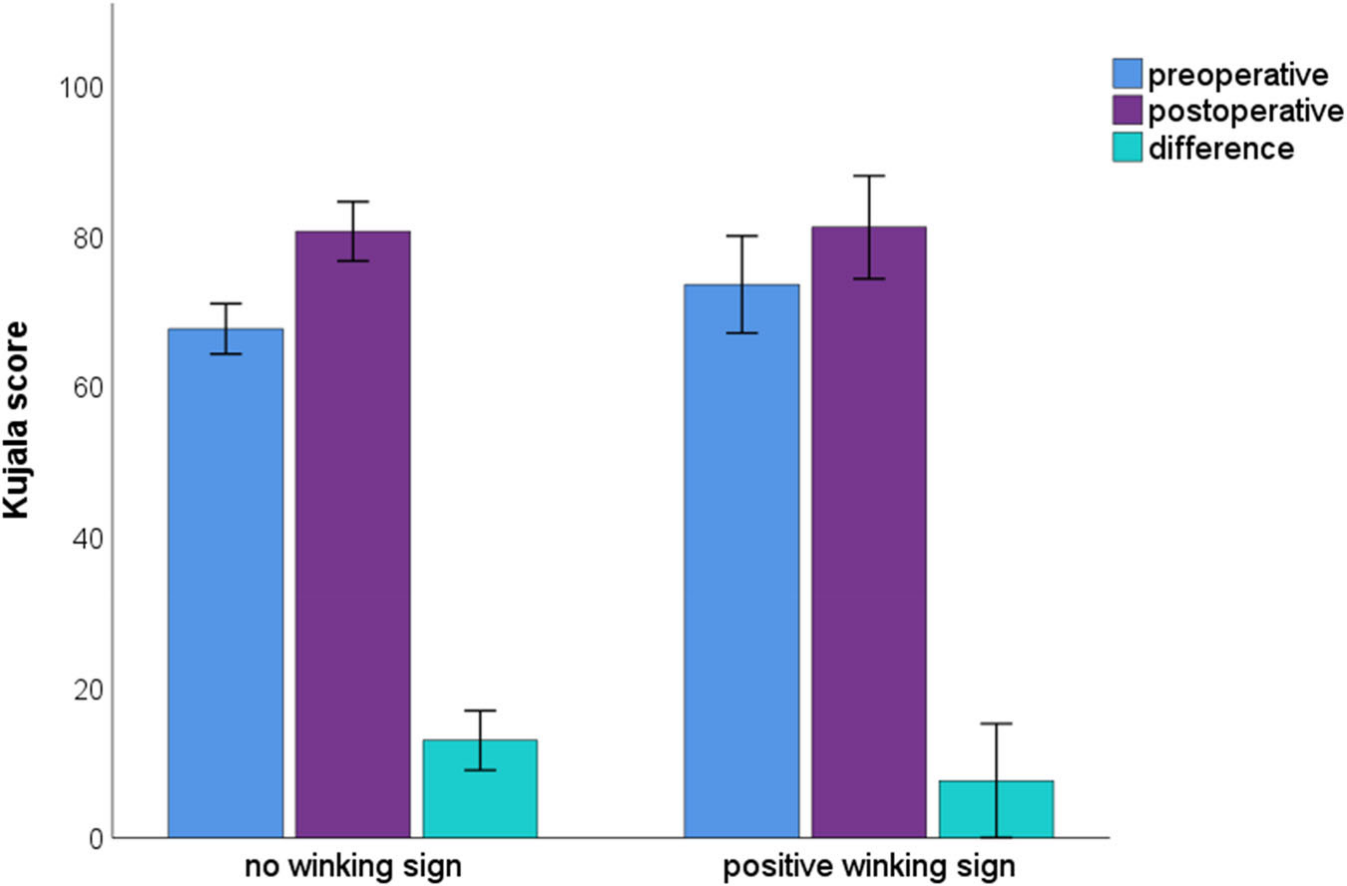
Kujala score preoperative, postoperative and the difference according to the presence of a winking sign.

Functional outcome scores showed an improvement from preoperative to postoperative evaluations, with an overall Kujala score increasing from 68.9 ± 16 to 80.8 ± 19 (*p* < 0.001). Patients with a positive winking sign demonstrated an improvement with a change in Kujala scores of 7.6 ± 18. Patients without a winking sign showed an improvement in the Kujala scores of 13.0 ± 20 (*p* = 0.170) (Table 2 and Figure 3).

**Table 2:** Patient‐reported outcome measurement.

	Positive winking sign (*n* = 24)	No winking sign (*n* = 97)	*p* value
Kujala preoperative	73.6 ± 15.3	67.5 ± 16.6	0.125
Kujala postoperative	81.3 ± 16.2	80.5 ± 19.6	0.770
Kujala delta	7.6 ± 18.0	13.0 ± 19.7	0.170
Tegner preoperative	3.7 ± 1.3	3.6 ± 1.4	0.899
Tegner postoperative	3.7 ± 1.3	3.4 ± 1.7	0.464
Tegner delta	0.0 ± 1.8	−0.5 ± 2.0	0.373

The overall demographic composition and preoperative deformity analyses yielded comparable results between the two groups (n.s.), except for increased femorotibial rotation (11.8° ± 7° vs. 8.5° ± 5°; *p* = 0.031) and elevated patellar tilt (31.6° ± 12° vs. 25.5° ± 10°; *p* = 0.006) in patients with a positive winking sign (Table 3).

**Table 3:** Preoperative deformity analysis.

	Positive winking sign (*n* = 24)	No winking sign (*n* = 97)	*p* value
Dysplasia type			0.494
A	2 (8.3%)	11 (11.3%)	
B	9 (37.5%)	29 (29.9%)	
C	6 (25%)	18 (18.6%)	
D	7 (29.2%)	29 (29.9%)	
None	0 (0%)	10 (10.3%)	
Femoral torsion (°)	23.4 ± 12.9	21.2 ± 11.4	0.504
Tibial torsion (°)	23.4 ± 8.9	24.5 ± 9.0	0.972
Femorotibial rotation (°)	11.8 ± 6.9	8.4 ± 5.4	**0.031**
HKA (°)	−0.8 ± 2.6	−1.1 ± 2.5	0.478
TT‐TG (mm)	17.4 ± 4.6	16.7 ± 4.2	0.258
Patella tilt (°)	31.6 ± 11.5	25.5 ± 9.6	**0.006**

*Note*: Significant values are marked in bold (*p* < 0.05).

Abbreviations: HKA, hip–knee–ankle angle, TT‐TG, tibial tuberosity–trochlear groove.

The distribution of surgical interventions was evenly balanced across the groups (n.s.), as shown in Table 4.

**Table 4:** Surgical treatment.

	Positive winking sign (n = 24)	No winking sign (n = 97)	*p* value
MPFL‐reconstruction	21 (87.5%)	92 (94.8%)	0.195
Trochleoplasty	20 (83.3%)	64 (66.0%)	0.099
Femoral osteotomy	5 (20.8%)	23 (23.7%)	0.763
TT‐medialization	8 (33.3%)	49 (50.5%)	0.131

Abbreviations: MPFL, medial patellofemoral ligament; TT, tibial tuberosity.

A total of 17% (4 out of 24) patients with a preoperative positive winking sign remained a positive winking sign in the postoperative follow‐up.

The interrater reliability for the measurements of the femorotibial rotation showed an ICC of 0.89, with a 95% confidence interval of 0.84 to 0.92, and a *p*‐value < 0.001. According to commonly used benchmarks [15], this indicates excellent reliability between the two raters. The categorical assessment of the winking sign showed a 100% agreement rate.

### Recurrent instability and complications

Only one patient (0.8%) without a winking sign experienced recurrent instability. Furthermore, the incidence of persistent apprehension (*n* = 4; 3.3%) and postoperative complications did not show significant differences between the groups.

## DISCUSSION

The primary aim of this study was to investigate the potential impact of preoperative femorotibial rotation on postoperative PROMs as well as functional outcomes such as recurrent instability and revision operations.

The most important finding of the present study was that a winking sign does not have a significant negative impact on postoperative patient‐reported outcome measurements. However, our results did reveal a trend that suggests a potential influence of the winking sign on these outcomes. While this trend was not statistically significant. Additionally, functional outcomes such as recurrent instability and the need for revision surgery showed no significant differences. This indicates that the presence of a winking sign preoperatively does not adversely affect these critical aspects of patient recovery.

This finding demonstrates that, despite undergoing patella stabilization surgery, a subset of patients maintained the winking sign after the procedure. It remains uncertain which specific aspects of the surgical technique might influence femorotibial rotation and contribute to this persistence. A recent study [12] suggest that femoral rotation alone does not have a significant impact on femorotibial rotation.

None of the patients in our study who retained a postoperative winking sign underwent tibial tubercle medialization. This observation suggests that tibial tubercle medialization might influence the persistence of the winking sign, potentially by altering knee joint mechanics and rotation. However, while the mean TT‐TG distance was slightly higher in the group with a positive winking sign, this difference did not reach statistical significance. Therefore, we did not identify a clear correlation between increased preoperative TT‐TG distance and the presence of the winking sign in our cohort. Another study demonstrated in a cadaver study that the TT‐TG distance was linearly dependent on knee joint rotation, changing by 0.52 mm for every degree of knee joint rotation [19]. This underscores the complexity of the factors involved in knee joint mechanics and the need to consider multiple variables when assessing surgical outcomes.

Additional research is needed to explore how various surgical techniques and individual patient characteristics may influence the persistence of the winking sign and overall surgical success. By addressing these questions, future studies could provide more insights into optimizing surgical approaches and improving patient outcomes.

Beyond the inherent limitations of its retrospective design, this study is further constrained by several additional factors. The most important limitation is the variability in the different surgical procedures performed to address patellofemoral instability. This variability introduces numerous factors that could influence the outcomes, which are not associated with the winking sign. Furthermore, the study is also limited by the loss to follow‐up due to the absence of PROMs.

In summary, our study demonstrates that increased femorotibial rotation, as indicated by the presence of a winking sign, does not significantly impact postoperative outcomes. Specifically, there was no substantial effect on PROM or functional outcomes following patella stabilization surgery. These findings suggest that other factors may play a more critical role in determining the success of patella stabilization procedures, and that increased femorotibial rotation should not be considered a major determinant of postoperative recovery and function. Although in this study, the different operative steps could not be evaluated individually. Further investigation is required to determine whether the presence of a winking sign influences the outcomes of specific surgical techniques. This additional research will provide a more comprehensive understanding and potentially enhance the surgical techniques and strategies used in patella stabilization procedures.

## CONCLUSION

Patients with increased femorotibial rotation can expect to have a similar functional outcome following patellofemoral stabilizing surgery without increased complications or persistent instability. A tendency for less functional improvement in these patients suggests that femorotibial rotation should be further evaluated to determine whether it should be treated independently during patellofemoral stabilization surgery.

## AUTHOR CONTRIBUTIONS

**Gabriele Cirigliano, Marc Schmid, Sandro Hodel and Sandro Fucentese:** Conceptualization. **Marc Schmid and Giuseppe Loggia:** Data collection. **Marc Schmid and Sandro Hodel:** Data analysis. **Andreas Flury and Sandro Fucentese:** Analysis verification. **Marc Schmid and Sandro Hodel:** Writing the first draft of the manuscript. All authors reviewed and edited the manuscript and approved the final version of the manuscript.

## CONFLICT OF INTEREST STATEMENT

Prof. Dr. Fucentese is a member of the ESSKA‐EKA osteotomy group and receives personal fees for being a consultant for Medacta SA, Storz, Zimmer‐Biomet, and Smith&Nephew. These financial activities are outside the submitted work. The remaining authors declare no conflict of interest.

## ETHICS STATEMENT

Ethical approval for this study was obtained from Zurich Cantonal Ethics Commission (2023‐01376). The study was conducted in accordance with the Declaration of Helsinki. All patients have signed a general consent form.

## Data Availability

The datasets used and analyzed during the current study are available from the corresponding author on reasonable request.
